# Degradation of aniline by the combined process of ultrasound and hydrogen peroxide (US/H_2_O_2_)

**DOI:** 10.1016/j.mex.2019.02.033

**Published:** 2019-03-05

**Authors:** Somayeh Rahdar, Chinenye Adaobi Igwegbe, Mozhgan Ghasemi, Shahin Ahmadi

**Affiliations:** a Department of Environmental Health, Zabol University of Medical Sciences, Zabol, Iran; b Department of Chemical Engineering, Nnamdi Azikiwe University, Awka, Nigeria

**Keywords:** Degradation of aniline by the combined process of hydrogen peroxide and ultrasound, Aniline, Hydrogen peroxide, Ultrasound, Aqueous solutions, Water treatment

## Abstract

Aniline is an aromatic hydrocarbon discharged into the environment through certain industrial effluents, which thereby contaminate water resources. In this study, the performance of an oxidizing agent, hydrogen peroxide (H_2_O_2_) with ultrasound (US) for the removal of aniline from its aqueous solution was examined. The treatability of contaminated effluent using US/H_2_O_2_ with a frequency of 50 Hz for the treatment of aniline-contaminated water was investigated. The effects of operational parameters such as H_2_O_2_ concentration (0.01–0.07 mol/L), initial aniline concentration (20–120 mg/L), contact time (15–90 min) and pH (3–11) on the degradation of aniline was examined. Optimal H_2_O_2_ concentration, initial aniline concentration and contact time were obtained as 0.01 mol/L, 20 mg/L, and 45 min. The degradation process was more efficient at pH of 3. Removal efficiency of 95.91% was achieved at these optimum conditions. The results indicate that the combined US and H_2_O_2_ process at optimal conditions can be applied for the degradation of aniline with great efficiency.

## Graphical abstract

**Figure:**
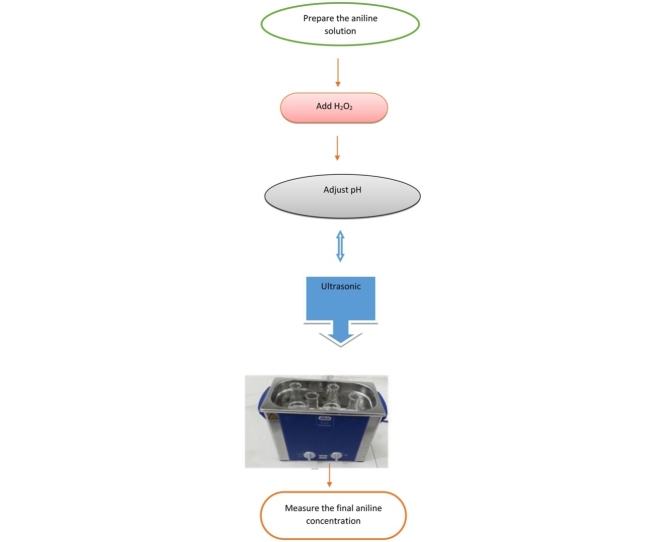

**Specifications Table**

**Table:** 

**Subject Area:**	Environmental Engineering
**More specific subject area:**	Pollutant degradation
**Method name:**	Degradation of aniline by the combined process of hydrogen peroxide and ultrasound
**Name and reference of original method:**	S. Rahdar, C.A. Igwegbe, A. Rahdar, S. Ahmadi, Efficiency of sono-nano-catalytic process of magnesium oxide nanoparticle in removal of penicillin G from aqueous solution, Desal. Water Treat. 106 (2018) 330–335 (Published) [9].
**Resource availability:**	N/A

**Table:** 

Protocol data
• The presented data demonstrated that the combined ultrasonic (US) and hydrogen peroxide (H_2_O_2_) process can be applied efficiently for the removal of aniline. • Data on the degradation kinetics, and the effect of operating parameters were provided. • The dataset will also serve as reference material to any researcher in this field.

## Method details

### Chemicals and materials

Aniline (C_6_H_5_NH_2_) of purity: 99.5% with a molecular weight: 93.13 g/mol and maximum adsorption (λ_max_): 280 nm, and 30% w/w hydrogen peroxide (H_2_O_2_) were purchased from Sigma-Aldrich Chemical Company (USA).A stock solution of aniline (concentration of 1000 mg/L) was prepared by dissolving the required amount in 1 L of deionized water. Other aniline concentrations used in the study were prepared from the stock solution. All chemicals used in this study were of analytical grade.

### Pilot ultrasonic

**Fig. 1:**
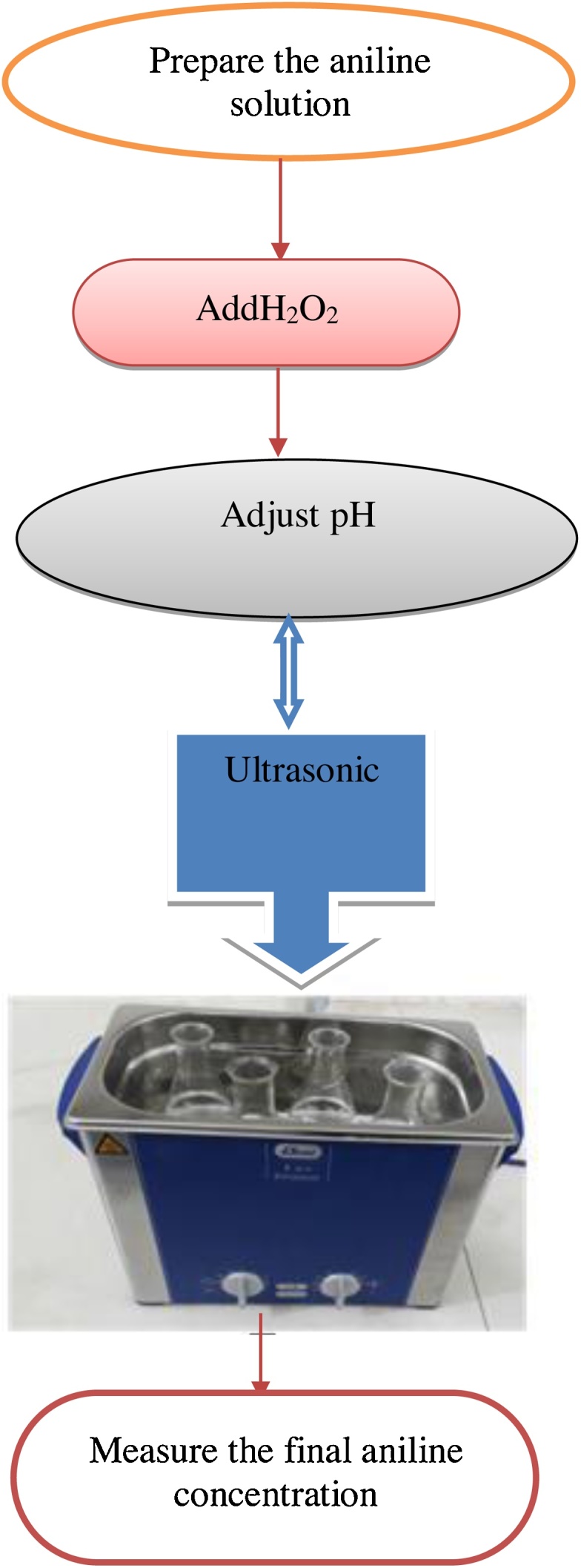
The schematic illustration of the sonochemical process.

Reactor of determined surface including a digital ultrasonic appliance that is made of Plexiglas with a volume of 3.7 L, input energy per unit of 2.5 W/cm^2^, and input power of 500 W including 100 mL of the water samples in the bath with ultrasonic (US) waves. The schematic illustration of the sonochemical process is shown in Fig. 1.

## Data analysis

The batch experiments were carried out to optimize aniline removal under different pH (3, 5, 7, 9 and 11), contact time (15, 30, 45, 60, and 90 min), H_2_O_2_ concentration (0.01, 0.04, and 0.07 mol/L), and initial aniline concentration (20, 40, 60, 80, and 120 mg/L). The pH of the aniline solution was adjusted by adding 0.1 mol/L hydrochloric acid (HCl) or 0.1 mol/L sodium hydroxide (NaOH) solutions. The pH was measured through a MIT65 pH meter. Each experiment involves preparing a 100 mL of aniline solution with a desired initial aniline concentration and varying the pH and H_2_O_2_ concentration. The sample was transferred into an ultrasonic bath (ElMA-Germany) generating supersonic waves at 240 W and 50 Hz. The samples were treated in the US bath at different contact time and the residual concentrations were measured afterward.

Aniline concentration was determined using a UV–vis spectrophotometer (Shimadzu Model: CE-1021-UK). The removal efficiency, R (%) was calculated based on the following formula [[1], [2], [3]]:

(1) $$\%R=\frac{\left(C_{0}-C_{f}\right)}{C_{0}}*100$$

Where *C*_0_ and *C_f_* represent the initial and final (after the degradation process) aniline concentrations, respectively.

## Effect of pH

**Fig. 2:**
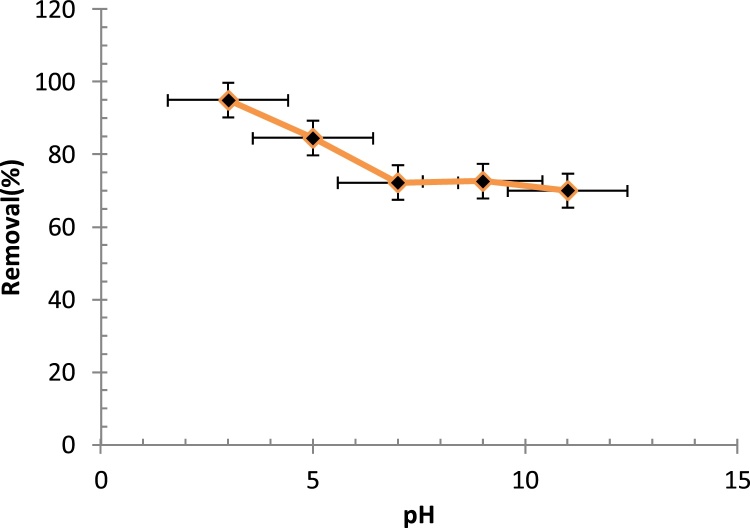
Effect of pH on the removal efficiency of aniline (Contact time: 60 min, H_2_O_2_ concentration: 0.01 mol/L, aniline concentration: 60 mg/L).

One of the most important parameters to be investigated in a chemical process is the pH of the reaction medium (Fig. 2). It affects the characteristics of the contaminant, the extent of decomposition of the organic matter and the efficiency of the degradation process. In order to determine the optimal pH, 100 mL samples were prepared and subjected to treatment in the chamber of the ultra-sonication device at different values of pH at a constant contact time of 60 min, and aniline and hydrogen peroxide (H_2_O_2_) concentrations of 40 mg/L and 0.01 mol/L, respectively. Maximum aniline removal efficiency was obtained at an acidic pH of 3, where the reaction between the hydroxyl radicals (^•^OH (and hydroxyl ions (OH^−^) ends up producing positive cavities at low pH values. On the other hand, molecular ionization of aniline was maximized in the acidic pH range due to the increased electrostatic attraction between the anionic and cationic species. At high pH values, OH^−^ contributes to the formation of dissolved compounds in the form of water [4], thereby hindering the adsorption process. Moreover, in an acidic media, the available amount (that is, stability) of ^•^OH is somewhat larger than that in a basic media, hence oxidation processes give better results in acidic media rather than basic ones, which is in agreement with the report by Zarrabi et al. [5].

## Effect of initial aniline concentration

**Fig. 3:**
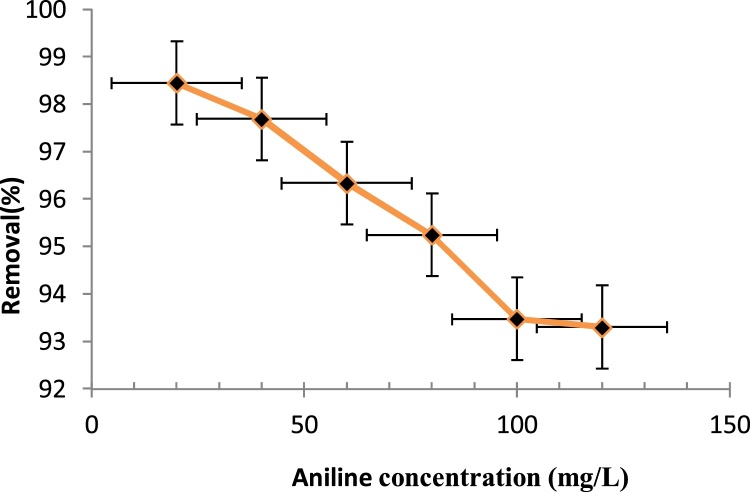
Effect of initial aniline concentration on the removal efficiency of aniline (Time: 45 min, H_2_O_2_ concentration: 0.01 mol/L, pH: 3).

In order to determine the optimum initial aniline concentration, the H_2_O_2_ concentration and pH were kept constant (Fig. 3). Since the number of hydroxyl radicals produced was constant, the rate of decomposition was reduced with higher aniline concentration, thereby lowering the removal efficiency [6], while at higher aniline concentrations (since H_2_O_2_ concentration and ultrasonic waves were unchanged), the ultrasonic waves did not reach the surfaces of all particles, reducing the extent to which the particles were oxidized [7]. In most studies on the oxidation of organic compounds, an increase in the concentration of the considered contaminant has been associated with a reduction on the removal efficiency [8].

## Effect of H_2_O_2_ concentration

**Fig. 4:**
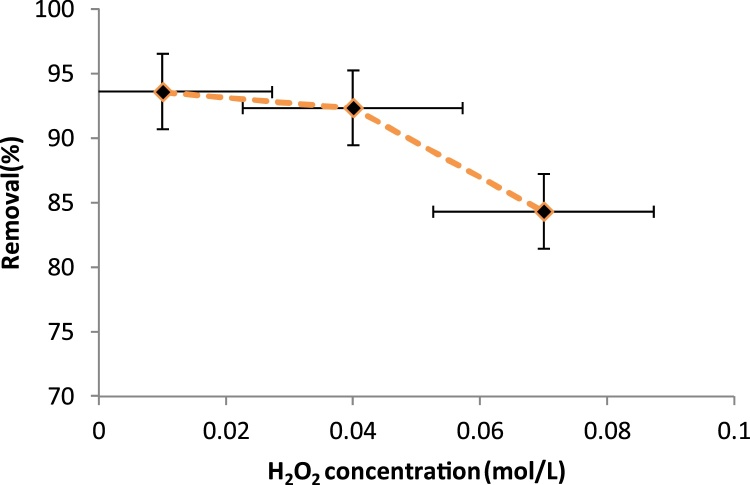
Effect of H_2_O_2_ concentration on the removal efficiency of aniline (Time: 45 min, aniline concentration: 20 mg/L, pH: 3).

Effect of H_2_O_2_ concentration on aniline removal was investigated at the optimum aniline concentration (20 mg/L) and the optimal pH of 3 (Fig. 4). The aniline removal efficiency decreased gradually from 93.58 to 84.3.9% as the concentration of H_2_O_2_ was increased from 5 to 15 mol/L. H_2_O_2_ plays an important role in the oxidation process where it serves as a source of ^•^OH. The technique includes H_2_O_2_ injection with the effluent in the reactor. During this process, ultrasound (US) is used to cleave the O—O bond in hydrogen peroxide and generate the hydroxyl radicals. The reactions describing the US/H_2_O_2_ process are shown in Eqs. (2), (3), (4), (5), (6), (7) [9]:

(2) H_2_O_2_ + US → 2HO

(3) H_2_O_2_ + HO^•^ → HO_2_^•^ + H_2_O

(4) H_2_O_2_ + HO_2_^•^ → HO^•^ + H_2_O + O_2_

(5) 2HO^•^ → H_2_O_2_

(6) 2HO_2_^•^ → H_2_O_2_+O_2_

(7) HO^•^ + HO_2_^•^ → HO_2_^•^ + O_2_

This could be attributed to the reaction between excessive H_2_O_2_ and ^•^OH according to the following equations and the formation of HO_2_^•^ which is on the negligible oxidative strength when compared to hydroxyl free radicals [10,11]. Therefore, the H_2_O_2_ concentration of 0.01 M was recognized as the optimal when the reaction rate was maximal. At higher concentrations, the H_2_O_2_ acted as an interfering agent and reacted with hydroxyl radicals, ^•^OH in the aqueous medium, inhibiting their attack to the contaminant molecules [12]. The higher the number of hydroxyl radicals, the higher the rate of decomposition and oxidation of the organic matter, and given that the number of radicals obtained from H_2_O_2_ of the hybrid system is higher than that of an isolated sonication system; the rate of oxidation will be higher in the hybrid system rather than the isolated sonication system [11].

## Effect of contact time

**Fig. 5:**
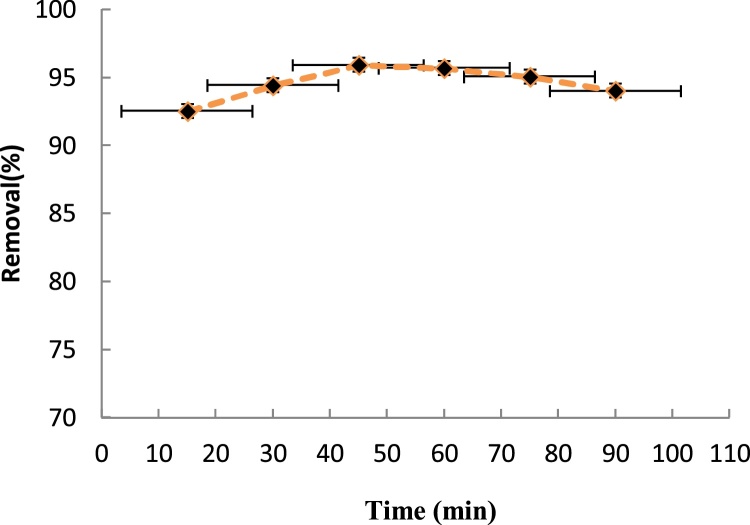
Effect of contact time on the removal efficiency of aniline (Aniline concentration: 20 mg/L, H_2_O_2_ concentration: 0.01 mol/L, pH: 3).

Higher removal efficiency was obtained with increasing contact time (while keeping all other conditions constant) (Fig. 5). Maximum removal efficiency was attained at a contact time of 45 min. Indeed, with lengthening the contact time, more hydroxyl free radicals were produced and contributed to the oxidation of the aniline molecules, thereby lowering the aniline concentration. Another explanation for the high rate of removal in the stage was the high concentration of aniline during this period, which enhanced the collisions between the aniline molecules and hydroxyl free radicals, thereby eliminating a larger amount of aniline. However, as time passed, aniline concentration became lower, so that the hydroxyl free radicals in the pilot were used to oxidize the aniline metabolites, lowering the removal rate [13,14].

## Degradation kinetics

The kinetic studies were carried out by taking decolorization into consideration under the optimum conditions for the process. To determine the characteristics of *m* and *b*, Eq. (8) was applied [15]:

(8) $$\frac{t}{1-(\frac{c}{c_{0}})}=m+b.t$$

Where *C*_0_ is the initial concentration of aniline (mg/L); *C* is the aniline concentration at time, t (mg/L); *m* and *b* are the two dimensionless characteristic constants of the model relating to the initial removal rate and maximum oxidation capacities, respectively.

**Fig. 6:**
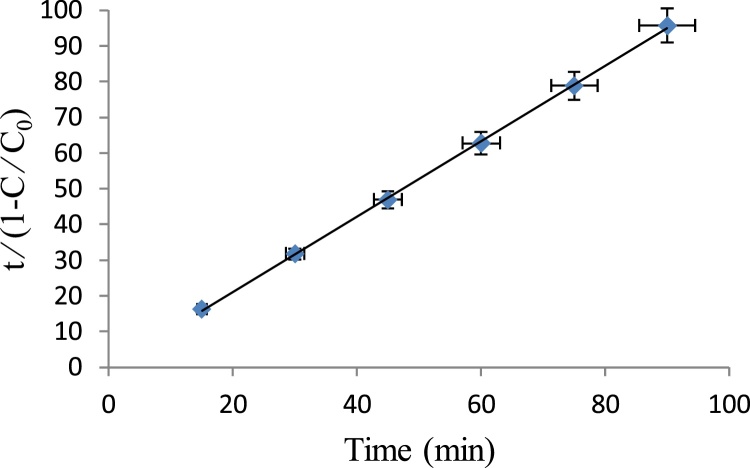
The relationship between *t*⁄ (1 − *C*⁄*C*_0_) and oxidation time, *t* at the optimum conditions for aniline removal.

A straight line was obtained by plotting *t*⁄ (1 − *C*⁄*C*_0_) against contact time, *t* (Fig. 6), where *m* and *b* were obtained from the slope and intercept of the straight line, respectively. According to the obtained results (Table 1 and Fig. 6), this study is more compatible with the degradation equation. The correlation coefficient (R^2^) of the degradation of aniline was high (R^2^ = 0.9997).

**Table 1:** The coefficients of determination and the characteristic constants of the kinetic model.

C_0_ (mg/L	Degradation			Pseudo-first-order	
	***b***	***m***	**R^2^**	***k_1_***	**R^2^**
20	1.0559	0.122	0.9997	0.0128	0.857

The loss of aniline was observed as a function of irradiation time and the experimental data were fitted to a pseudo-first-order rate model according to the following equation [15,16]:

(9) $$\ln\left(\frac{c}{c_{0}}\right)=-k_{1}t$$

**Fig. 7:**
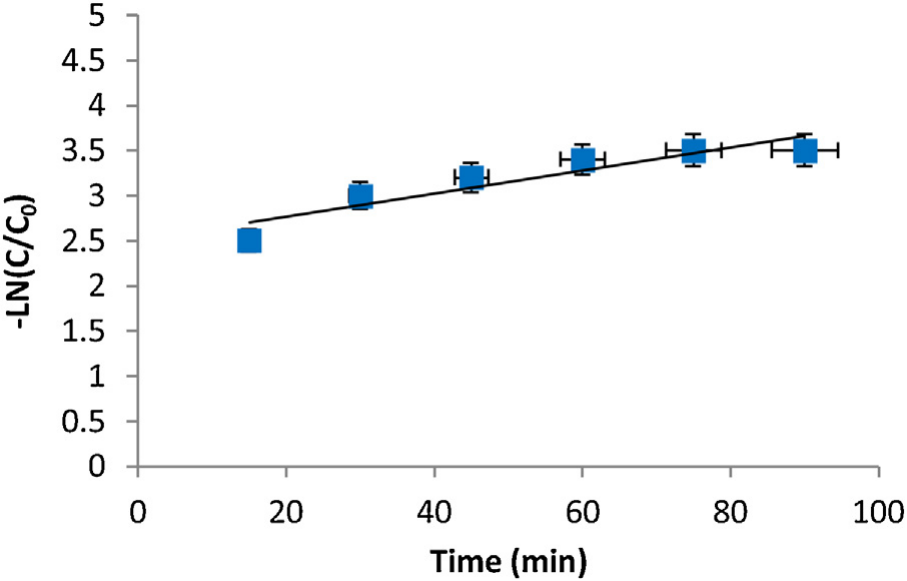
Pseudo-first-order plot for the degradation of aniline by US/H_2_O_2_.

Where *C*_o_ denotes the initial concentration in milligrams per liter, and *C* is the concentration value in milligrams per liter at time, t. The slope of the plot of *Ln* $\left(\frac{c}{c_{0}}\right)$ versus time (Fig. 7) gives the value of the rate constant, *k*_1_ (min^−1^). The process follows the pseudo-first-ordermodel (R^2^ = 0.857) (Table 1).

## Conclusion

The degradation process was more efficient at pH of 3. Maximum removal efficiency of 95.91% was reached at H_2_O_2_ concentration of 0.01 mol/L, initial aniline concentration of 20 mg/L, and contact time of 45 min. The results reveal that the combined ultrasound and hydrogen (US/H_2_O_2_) process can be applied for the effective degradation of aniline.

## Funding sources

This paper is the result of the approved project at Zabol University of Medical Sciences, Zabol, Iran.

## Conflict of interests

The authors declare to have no conflict of interests.
